# Kaempferide Prevents Photoaging of Ultraviolet-B Irradiated NIH-3T3 Cells and Mouse Skin via Regulating the Reactive Oxygen Species-Mediated Signalings

**DOI:** 10.3390/antiox12010011

**Published:** 2022-12-21

**Authors:** Jong-Kyu Choi, Oh-Yun Kwon, Seung-Ho Lee

**Affiliations:** Department of Nano-Bioengineering, Incheon National University, 119 Academy-ro, Incheon 22012, Republic of Korea

**Keywords:** kaempferide, UVB, photoaging, ROS, MAPK, AKT

## Abstract

Kaempferide (KFD) is a naturally occurring flavonoid that exists in various medicinal plants. The pharmaceutical properties of KFD, including its anti-cancer, antioxidant and anti-diabetic effects, have been noted, but the effects of KFD on photoaging and their underlying molecular mechanism have yet to be elucidated. In this study, we investigated the effects of KFD on Ultraviolet-B (UVB)-mediated photoaging processes using in vitro and in vivo photoaging model systems. The topical administration of KFD on mouse dorsal areas suppressed UVB-mediated wrinkle formation and epidermal thickening. In addition, the UVB-mediated reduction of dermal collagen content, which was estimated by Masson’s trichrome staining, was recovered through KFD treatments. Furthermore, we found that UVB-induced abnormal values of procollagen type-1 (COL1A1), metalloproteinases (MMP-1a and MMP-3) and proinflammatory cytokines (IL-8, MCP-3 and IL-6) on mouse skin tissue as well as NIH-3T3 cells was recovered through KFD treatment. The administration of KFD to NIH-3T3 cells suppressed the UVB-mediated upregulation of reactive oxygen species (ROS), mitogen-activated protein kinases (MAPKs) and AKT phosphorylation. Furthermore, the treatment of ROS inhibitor restored the UVB-induced MAPKs and AKT phosphorylation as well as the abnormal expression of photoaging related genes. These findings indicate that KFD can attenuate UVB-induced ROS elevation to elicit anti-photoaging activity. Taken together, our data suggest that KFD could be developed as a potential natural anti-photoaging agent.

## 1. Introduction

As environmental pollution increases and destroys the stratospheric ozone, the increase in high-wavelength ultraviolet-A and B (UVA and UVB) radiation on Earth is a serious environmental threat and public health problem. This is because the skin is one of the most important organs of the human body, since it plays a protective role as a barrier against detrimental factors, such as viruses and toxins [1,2]. Photoaging, which is induced by chronic overexposure to UVB, is generally associated with wrinkle formation, reduced elasticity, dyspigmentation and loss of subcutaneous fat [3,4,5]. In addition, excessive exposure to UV radiation can increase dermatological concerns, since UVB can promote metabolic disease and skin cancer [6,7,8]. Therefore, the demand for more effective and secure anti-photoaging agent development is gradually increasing.

The reduction of dermal collagen content in UVB-irradiated skin is recognized as a major photoaging process. UVB irradiation of the skin reduces procollagen type-1 (COL1A1), which stimulates dermal collagen synthesis [9,10]. In addition, the expression of metalloproteinases (MMPs), which mediate the degradation of dermal collagen, is induced by UVB exposure to the skin [11,12]. Therefore, UVB mediates the abnormal expression of photoaging-related genes, such as COL1A1 and MMPs, which are considered to be a major cause of the photoaging processes.

Excessive exposure of UVB to the skin can induce inflammatory responses, which are recognized as a cause of acute edema and erythema [13]. The UVB-mediated overproduction of proinflammatory cytokines, such as IL-8, MCP-3 and IL-6, was suggested as an important mediator in UVB-mediated loss of subcutaneous (SC) fat [14]. Therefore, photoaging-related genes, such as COL1A1, MMPs IL-6, IL-8 and MCP-3, have been recognized as therapeutic targets.

It has been known that UVB radiation stimulates the production of reactive oxygen species (ROS), which can activate various intracellular signaling pathways. Excessive ROS generation by UVB irradiation induces cellular oxidative stress, leading to inflammatory damage and accelerates photoaging in skin through regulating the gene expression associated with biological changes elicited by UVB [15].

Mitogen-activated protein kinases (MAPKs) and protein kinase B (AKT) have been shown to be a key intracellular regulator in several disease processes, such as inflammation, apoptosis and photoaging [16,17,18,19]. UVB-induced ROS accumulation activates MAPKs and AKT signaling, and is critical in regulating the expression of disease-related genes, which is important in halting disease progression. In fact, UVB irradiation induces ROS generation, sequentially activating MAPKs and AKT in the skin, resulting in the abnormal expression of photoaging-related genes, such as COL1A1 and metalloproteinases (MMPs) [10,20,21,22]. It was reported that oral administration of p38 MAPK inhibitor could attenuate UVB-mediated photoaging processes [13]. These reports provide evidence that ROS-mediated signaling pathways act as key regulators in UVB-mediated photoaging processes and could be potential therapeutic targets in the development of anti-photoaging agents.

Since there are many plant-based Asian medicines, much attention has been focused on their single constituents, such as phenolic acids and flavonoids. Kaempferide (3, 5, 7-trihydroxy-4′-methoxyflavone, KFD) is a naturally detected flavonoid that is a major constituent of the *Alpinia officinarum* rhizome [23]. The pharmacological and biological properties of KFD have been reported, showing that it has antioxidant [24,25], anti-cancer [26,27], anti-inflammation [23,25], anti-adipogenic [28] and anti-obesity [29] activities. However, the anti-photoaging activity of KFD has never been elucidated.

Interestingly, in our previous study, water extracts of the *Alpinia officinarum* rhizome (WEAOR) were revealed to have strong anti-photoaging activity, and KFD was determined to be a major constituent of WEAOR [30]. Based on these results, in this study, to determine whether KFD could be a functional single constituent of WEAOR, which has strong anti-photoaging activity, we evaluated the effects of KFD on UVB-induced photoaging processes in mice skin as well as NIH-3T3 skin fibroblast cells and investigated the underlying molecular mechanism of KFD-mediated anti-photoaging processes.

## 2. Materials and Methods

### 2.1. Materials

Kaempferide was obtained from the TOKYO chemical industry (TCI, Tokyo, Japan). SYBR^®^ Green Realtime PCR Master Mix was obtained from Toyobo (Tokyo, Japan), and TRIzol^®^ Reagent was obtained from Invitrogen (Waltham, MA, USA). Rabbit anti-phospho-MAPKs (ERK: 9102S, p38: 9212S, JNK: 9251S), rabbit anti-MAPKs (ERK: 9101S, p38: 9211S, JNK: 9252S) and rabbit anti-phospho-AKT (4060S) and rabbit anti-AKT (9272S) were obtained from Cell Signalling Technology, Inc. (Danvers, MA, USA). Rabbit anti-COL1A1 (PA5-29569) and rabbit anti-MMP-1a (PA5-27210) were obtained from Invitrogen (Middlesex County, MA, USA). N-acetyl-L-cysteine (NAC) (A9165) was purchased from Sigma-Aldrich Co. (St. Louis, MO, USA).

### 2.2. Animal Experiments

Six-week-old C57BL/6 (female) mice were purchased from Raonbio Inc. (Seoul, Korea). Animals were habituated for one week in a pathogen-free animal house under a 12 h light/dark cycle at a constant temperature of 23 ± 2 °C, with 50 ± 10% humidity. Mice were randomly divided into the following four groups: non-treated (control, *n* = 6), UVB-irradiated (UVB, *n* = 6), UVB-irradiated with pre-treatment using KFD 250 μM (UVB + KFD250, *n* =6) and UVB-irradiated with pre-treatment using KFD 500 μM (UVB + KFD500, *n* = 6). The KFD was dissolved in glycerol: ethanol: H2O (5:3:2). Each solution (100 µL) was treated to the dorsal region of the mice skin every other day for 10 weeks. UVB was irradiated using a BIO-LINK 312 UVB irradiation system (VILBER Co., Seoul, Republic of Korea). The dorsal hair of the mice was shaved once a week. The mice were exposed to UVB between one MED (one MED = 50 mJ/cm^2^) and six MED every other day for 10 weeks, and the total amount of UVB irradiation was 94.5 MED (4725 mJ/cm^2^). One MED is defined as the minimum dose of radiation required to form an erythema with sharp margins after 24 h. After finishing 10 weeks of UVB exposure, the mice’s skin was photographed and the number of wrinkles was counted under a digital microscope (Nikon, Tokyo, Japan).

### 2.3. H&E and Masson’s Trichrome Staining

At the end of the experiments, the skin tissues were isolated, fixed in 10% paraformaldehyde solution and embedded in paraffin. The skin tissue was sliced (5 μm thick) and visualized by hematoxylin and eosin (H&E) staining. Masson’s trichrome staining was used to reflect the density of collagen fibers [7]. The stained slides were photographed, and the thickness of the epidermis and the change of lipid droplets were measured using the ImageJ program. The collagen fiber content, which was stained blue in Masson’s trichrome staining, was converted to black, and the intensity of the blackness was estimated using the ImageJ program.

### 2.4. Cytotoxicity Analysis

Mouse skin fibroblast cells (NIH-3T3) purchased from the Korean Type Culture Collection (KTCC, Seoul, Republic of Korea) were maintained in Dulbecco’s Modified Eagle’s Medium (DMEM, HyClone, UT, USA) with penicillin (100 units/mL), streptomycin (100 μg/mL) and fetal bovine serum (10%, Corning, NY, USA). To estimate the cytotoxicity of KFD, 1 × 10^4^ cells of NIH-3T3 cells were cultured in a cell culture plate (96-well) and further incubated for 24 h in a CO_2_ incubator maintained at a constant temperature of 37 °C. After replacing the culture media with serum-free DMEM, KFD (0~100 μM) was added to each well and further incubated for 24 h. WST-1 solution (10 µL/well) (DoGenBio Co., Seoul, Republic of Korea) was added to each well and further incubated during an additional hour in a CO_2_ incubator. Cell viability was calculated by measuring the absorbance at 450 nm using a microplate reader (iMark Microplate Reader, Bio-Rad Laboratories, Inc. Hercules, CA, USA).

### 2.5. Irradiation of UVB

NIH-3T3 cells (2 ×10^5^/well) were cultured in a cell culture plate (six-well) during 24 h at 37 °C in a CO_2_ incubator. After replacing the culture media with serum-free DMEM, KFD was added and further incubated for 24 h. After washing with phosphate buffered saline (PBS), NIH-3T3 cells covered with 1 mL of PBS were irradiated by UVB (25 mJ/cm^2^) using a BIO-LINK 312 UVB irradiation system (VILBER Co., Seoul, Republic of Korea). The cell culture media was then changed to complete media (DMEM containing 100 units/mL of penicillin, 100 μg/mL of streptomycin and 10% of FBS), incubated during 24 h in a CO_2_ incubator and used for the next assays.

### 2.6. Reactive Oxygen Species (ROS) Assessment

At the end of UVB-irradiation to NIH-3T3 cells, each well was washed with PBS and then 2′–7′dichlorofluorescin (DCFH-DA) solution (30 μM, Sigma-Aldrich Co., St. Louis, MO, USA) was added and further incubated for one hour at 37 °C in a CO_2_ incubator. After washing with PBS two times, 100 μL of PBS was then added to each well and the intensity of fluorescence was estimated by using a microplate fluorometer (Tecan, Mannedorf, Switzerland).

### 2.7. Quantitative Real-Time Polymerase Chain Reaction (qRT-PCR)

The total RNA of mouse skin tissue and NIH-3T3 cells was extracted by using TRIzol^®^ Reagent. One microgram of total RNA and 10 pM of oligo dT primer were used to synthesize complementary DNA (cDNA). SYBR^®^ Green Realtime PCR Master Mix was used for qRT-PCR performed in a RT-PCR instrument (CFX ConnectTM, Bio-Rad Co., Hercules, CA, USA). The relative expression of each gene was evaluated using the Ct method, and it was normalized with the value of glyceraldehyde 3-phosphate dehydrogenase (GAPDH). The sequences of primer used in this study are listed in Table 1.

### 2.8. Western Blot Analysis

The NIH-3T3 cells were lysed using a Tris-based protein lysis buffer [30] on ice for 1 h. After centrifuging (13,000× *g*, 4 °C, 15 min), the supernatants were used for Western blotting. Twenty micrograms of lysates were electrophoresed in sodium dodecyl sulfate (SDS) polyacrylamide gel and transferred to nitrocellulose membranes. After blocking the nonspecific signals by incubating with blocking buffer [30], the membranes were reacted with each primary antibody (1:1500) during 12–14 h at 4 °C. After washing with tris-buffered saline with tween 20 (TBS-T), horseradish peroxidase (HRP) conjugated rabbit secondary antibody (1:3000) (Santa Cruz, Dallas, TX. USA) was added and incubated at room temperature for 2 h. Each protein band was visualized using an enhanced chemiluminescence (ECL) detection kit (Bio-Rad, Hercules, CA, USA).

### 2.9. Statistical Analysis

All experiments were conducted in triplicate and repeated at least three times. A two-tailed, unpaired Student’s *t*-test and analysis of variance (ANOVA) followed by Tukey’s post hoc test using Prism 5 software (Graph-Pad Software, Inc., San Diego, CA, USA) were used for statistical analysis, and *p* < 0.05 was considered to be statistically significant.

## 3. Results

### 3.1. Administration of Kaempferide (KFD) Inhibited UVB-Induced Wrinkle Formation and Epidermal Thickening in Mice Skin

To determine whether KFD has anti-photoaging activity or not, at first, KFD was topically administrated to the dorsal area of UVB-irradiated mice for 10 weeks, and wrinkle formation and epidermis thickness were compared with that of a control mice group (without treatment with KFD).

**Figure 1:**
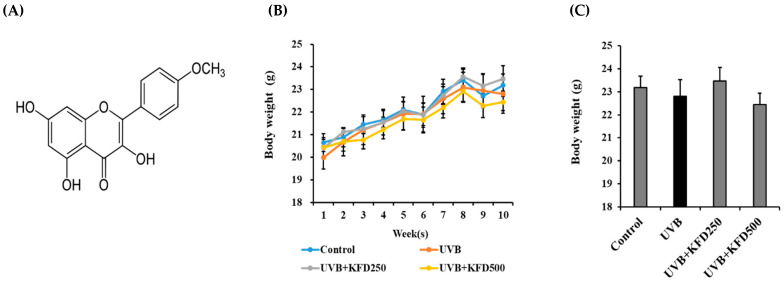
The topical administration of kaempferide (KFD) did not affect body weight. (**A**) Chemical structure of the KFD used in this study. (**B**) Body weight changes of each mouse group over 10 weeks (*n* = 6). (**C**) Body weight of each mouse group at the end of the experiments (*n* = 6). All data are expressed as the mean ± standard error of the mean (SEM). There were no significant differences between groups.

**Figure 2:**
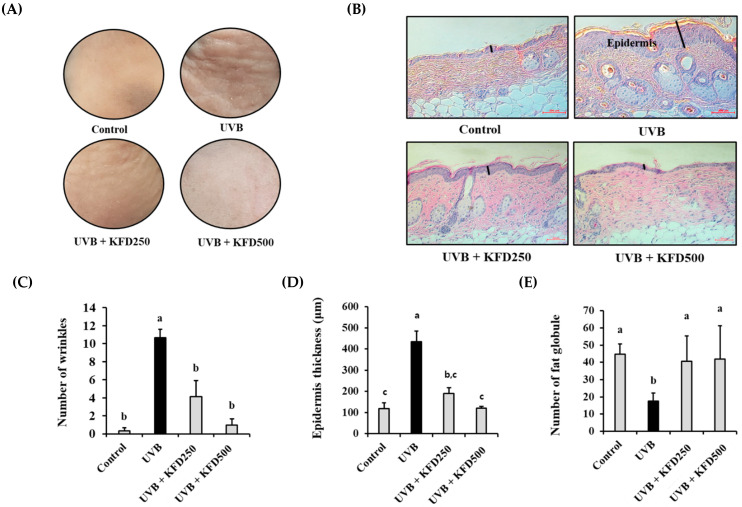
Topical administration of kaempferide (KFD) attenuated UVB-induced wrinkle formation in mice skin. (**A**) The skin of each mouse group (*n* = 6) was photographed at the end of the experiment and (**C**) the number of wrinkles was estimated under a microscope. (**B**) The skin of each mouse group (*n* = 6) was isolated at the end of the experiments and stained with H&E solution. A black line on the picture indicates the thickness of the epidermis. (**D**) The epidermal thickness was estimated using the ImageJ program, and (**E**) the number of subcutaneous fat globules was counted under a microscope. Scale bar: 200 µM. All values are expressed as the mean ± standard deviation (SD). Different letters (a–c) indicate the significant differences between groups (*p* < 0.05).

The topical administration of KFD to the mice’s dorsal areas for 10 weeks did not affect body weight (Figure 1), but the number of wrinkles in KFD-treated mice groups (KFD250: 4.16 ± 4.26, KFD500: 1.00 ± 1.67) was significantly (*p* < 0.05) reduced when compared with the UVB-irradiated mice group (10.66 ± 2.33) (Figure 2A). In addition, we found that UVB-mediated epidermal thickness was significantly (*p* < 0.05) decreased in KFD-treated mice groups (KFD250: 190.47 ± 45.49, KFD500: 119.95 ± 13.69) when compared with that of the UVB mice group (433.11 ± 89.15) (Figure 2B,D). Furthermore, we found that the number of fat globules, which is an indicator of UVB-irradiated skin aging, was decreased in UVB-treated mice (17.50 ± 4.76) when compared with that of the control group (44.66 ± 6.08), but it was restored in KFD-treated mice skin (KDF250: 40.6 ± 14.69, KFD500: 42.00 ± 19.20) (Figure 2B,E). These results suggest that the topical administration of KFD could effectively inhibit UVB-mediated photoaging processes.

### 3.2. KFD Administration Attenuated the UVB-Mediated Disappearance of Collagen Content in Mouse Skin

**Figure 3:**
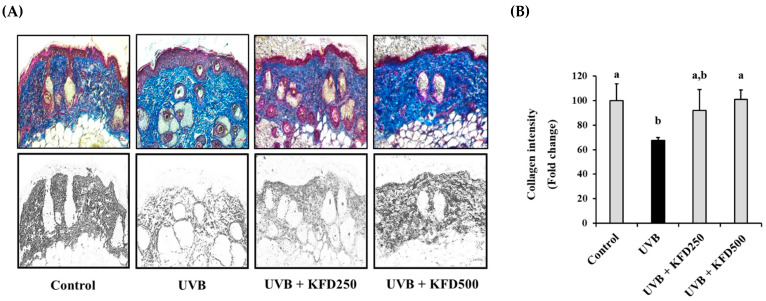
UVB-induced loss of epidermal collagen content was attenuated by kaempferide (KFD). (**A**) The skin of each mouse group (*n* = 6) was isolated at the end of the experiments and stained with Masson’s trichrome staining solution (upper panel), and the collagen contents, which were stained blue, were converted to black using the ImageJ program (lower panel). Scale bar: 100 µM. (**B**) The intensity of the collagen contents was estimated using the ImageJ program and tabulated. All data are expressed as the mean ± standard deviation (SD). Different letters (a and b) indicate the significant differences between groups (*p* < 0.05).

Since the collagen contents in the dermis area of the skin are important in maintaining the skin’s strength against photoaging processes, we then evaluated the effects of KFD on the change in collagen contents in UVB-irradiated mouse skin. After finishing the experiment, the contents of collagen fibers were visualized by Masson’s trichrome staining; collagen contents stained blue were converted to black and then estimated using the ImageJ program. As shown in Figure 3, UVB-irradiated mice showed a decreased intensity of collagen (67.70 ± 2.09) when compared with that of the control group (100 ± 13.76). However, it was significantly (*p* < 0.05) recovered in KFD-treated mouse skin (KFD250: 91.83 ± 17.23, KFD500: 101.08 ± 7.65). To further elucidate the effects of KFD on UVB-mediated changes in collagen contents in mice skin, the expression of procollagen type-1 (COL1A1), which has a role in collagen synthesis and metalloproteinases (*MMP-1a* and *MMP-3*) genes, which are important in the degradation of collagen, was estimated by qRT-PCR.

**Figure 4:**
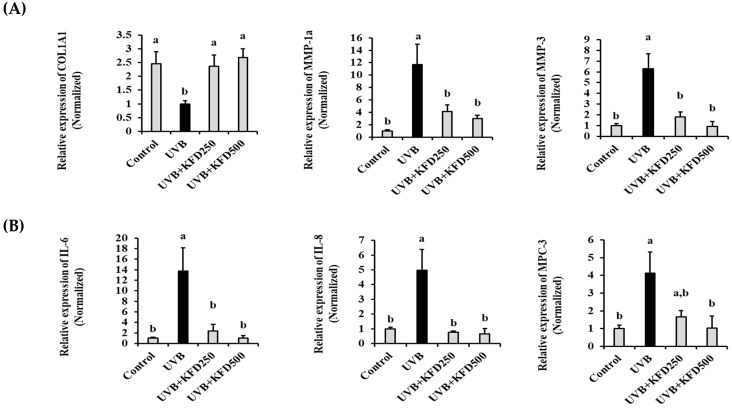
UVB-induced expression of photoaging-related genes was recovered by kaempferide (KFD) administration. The value of (**A**) *COL1A1*, *MMP-1a*, and *MMP-3*, (**B**) *IL-6, IL-8* and *MCP-3* in each mice group (*n* = 6) was estimated by qRT-PCR. All data are expressed as the mean ± standard deviation (SD). Different letters (a and b) indicate the significant differences between groups (*p* < 0.05).

As shown in Figure 4A, the expression of *COL1A1* on the UVB-irradiated mouse skin was decreased when compared with that of the control group (non-treated) (*p* < 0.05), but it was significantly recovered in KFD-treated mice skin. On the other hand, the UVB-induced expression of *MMP-1a* and *MMP-3* was dramatically increased in the UVB mice group when compared with that of the control mice group, and it was significantly (*p* < 0.05) decreased in KFD-administrated mice skin. These results suggest that the preventive effects of KFD on the UVB-induced reduction of collagen contents in skin could be a result of restoring the UVB-mediated abnormal expression of *COL1A1, MMP-1a* and *MMP-3* expression.

### 3.3. The UVB-Induced Overexpression of Proinflammatory Cytokines Was Attenuated by KFD Administration

Chronic exposure to UVB often induces an inflammatory response in the skin. In addition, the UVB-mediated overexpression of proinflammatory cytokines, such as MCP-3, IL-8 and IL-6, is considered a cause of the reduction of subcutaneous fat, which is a major phenotype in photoaging processes. Therefore, the inhibitory effects of KFD on the UVB-induced expression of proinflammatory cytokines were then investigated. As shown in Figure 4B, the UVB-induced expression of *IL-6, IL-8* and *MCP-3* on mouse skin was dramatically increased when compared with that of the control mice group. However, that expression was significantly attenuated in the KFD-treated mice skin. These results suggest that KFD could be used for attenuating the abnormal expression of pro-inflammatory cytokines, such as IL-6, IL-8 and MCP-3 induced by UVB irradiation.

### 3.4. Ultraviolet-B Mediated Overproduction of Reactive Oxygen Species (ROS) Was Inhibited by KFD Treatment

**Figure 5:**
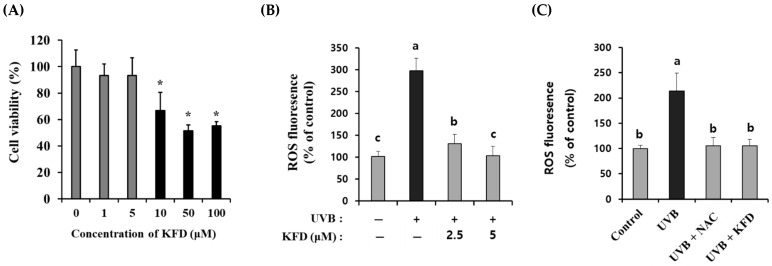
Administration of non-toxicological levels of KFD attenuated the UVB-induced ROS generation in NIH-3T3 skin fibroblast cells. (**A**) KFD was administered to NIH-3T3 cells for 24 h, and the cell viability of the NIH-3T3 cells was estimated with a WST-assay kit. (**B**) UVB-induced ROS production was dose dependently attenuated by KFD treatment in NIH-3T3 skin fibroblast cells. (**C**) UVB-induced ROS generation was prevented by treatment with NAC, a ROS inhibitor, in NIH-3T3 skin fibroblast. The data shown are representative of at least three independent experiments. All data are expressed as the mean ± standard deviation (SD) of three wells. Different letters (a–c) indicate the significant differences between groups (*p* < 0.05) and * indicates significant differences when compared to the control; * = *p* < 0.05.

To further evaluate the effects of KFD on UVB-mediated photoaging processes, the cellular toxicity of KFD on NIH-3T3 cells was first estimated. As shown in Figure 5A, the cell viability of NIH-3T3 cells did not change when KFD was treated with a concentration of 5 µM. Based on this result, non-toxicological levels of KFD (2.5 µM and 5 µM) were used to estimate the anti-photoaging properties. Based on this result, non-toxicological levels of KFD (2.5 µM and 5 µM) were used to estimate the anti-photoaging properties. As shown in Figure 5B, ROS overproduction induced by UVB-irradiation was dose dependently inhibited (*p* < 0.05) by non-toxicological levels of KFD (2.5 µM and 5 µM) treatment in NIH-3T3 cells. Furthermore, KFD (5 µM) showed equivalent efficacy compared to NAC (1 mM), a ROS inhibitor, in attenuating the UVB-induced ROS overproduction (Figure 5C). Taken together, these results suggest that KFD could be used for diminishing the oxidative stress induced by UVB-irradiation in NIH-3T3 skin fibroblast cells.

### 3.5. KFD Restored the Abnormal Expression of Photoaging-Associated Genes in NIH-3T3 Cells

**Figure 6:**
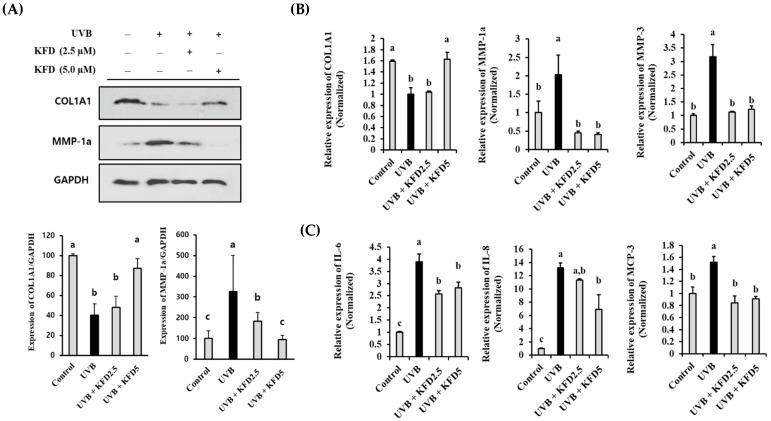
UVB-mediated expression of photoaging related genes on NIH-3T3 cells was attenuated by kaempferide (KFD) administration. NIH-3T3 cells were irradiated with UVB with and without KFD (2.5 µM: KFD2.5 and 5 µM: KFD5), and the expression of (**A**) COL1A1 and MMP-1a was evaluated by Western blotting. (**B**) Transcriptional differences of *MMP-1a, MMP-3, COL1A1*, (**C**) *MCP-3, IL-6* and *IL-8* genes were estimated by using qRT-PCR. Representative results of at least three independent experiments are shown. All data are expressed as the mean ± standard deviation (SD) of three wells. Different letters (a–c) indicate the significant differences between groups (*p* < 0.05).

As shown in Figure 6A, the administration of non-toxicological levels of KFD (up to 5 µM) recovered the downregulation of COL1A1 and increased the MMP-1a protein on the NIH-3T3 cells irradiated with UVB. These findings were further confirmed by using qRT-PCR. The UVB-mediated abnormal expression of *COL1A1*, *MMP-1a* and *MMP-3* genes was restored by KFD treatments in NIH-3T3 cells (Figure 6B). In addition, we found that the UVB-mediated increase of proinflammatory cytokine genes (*IL-6, IL-8* and *MCP-3*) was significantly (*p* < 0.05) attenuated by KFD treatment in NIH-3T3 cells (Figure 6C). These results suggest that administration of non-toxicological levels of KFD could effectively attenuate the UVB-mediated abnormal expression of COL1A1, MMPs and proinflammatory cytokines (MCP-3, IL-8 and IL-6) in NIH-3T3 cells.

### 3.6. KFD Prevented the UVB-Induced Photoaging Events through Attenuating Mitogen-Activated Protein Kinases (MAPKs) and AKT Activation

**Figure 7:**
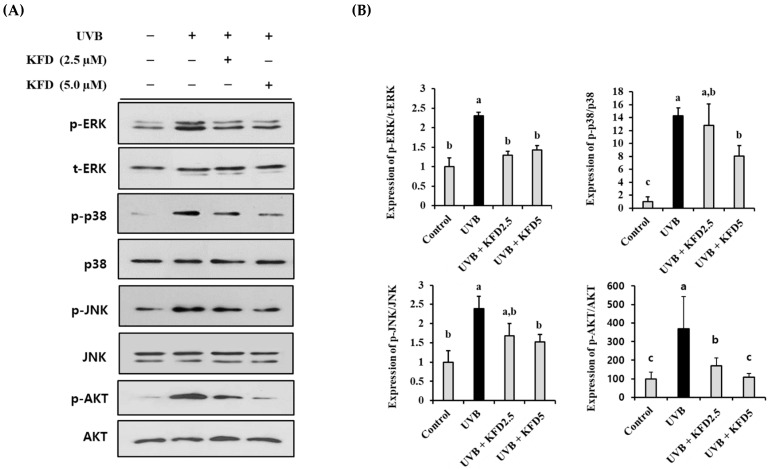
UVB-induced phosphorylation of MAPKs and AKT was attenuated by kaempferide (KFD) treatment. NIH-3T3 cells were irradiated with UVB with and without KFD (2.5 µM: KFD2.5 and 5 µM: KFD5) and the expression of (**A**) MAPKs (p-ERK/ERK, p-p38/p38, p-JNK/JNK) and p-AKT/AKT was estimated by Western blotting. (**B**) The density of each band of Western blotting was estimated by the ImageJ program, and tabulated representative results of at least three independent experiments are shown. All data are expressed as the mean ± standard deviation (SD). Different letters (a–c) indicate significant differences between groups (*p* < 0.05).

To determine the intracellular regulator in KFD-mediated anti-photoaging events, the effects of KFD on the UVB-induced change of MAPKs and AKT were investigated. As shown in Figure 7, UVB-induced phosphorylation of MAPKs (ERK, p38 and JNK) and AKT was significantly (*p* < 0.05) attenuated by KFD treatments.

**Figure 8:**
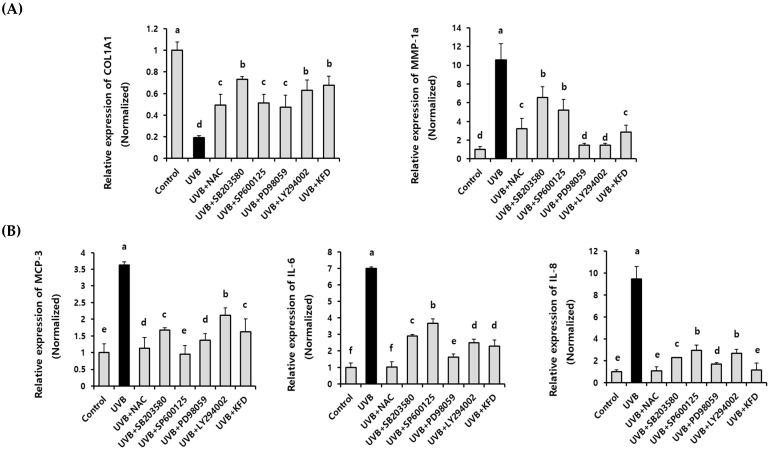
UVB-induced expression of photoaging-related genes was restored by the administration of MAPK inhibitors as well as kaempferide (KFD). NIH-3T3 cells were irradiated with UVB in the presence or absence of either ROS inhibitor: NAC, MAPK inhibitors (SB203580: p38 inhibitor, SP600125: JNK inhibitor, and PD98059: MEK inhibitor), PI3K inhibitor: LY294002, or KFD (5 µM). The value of (**A**) MMP-1a, COL1A1, (**B**) MCP-3, IL-8 and IL-6 was estimated by qRT-PCR. Representative results of at least three independent experiments are shown. All data are expressed as the mean ± standard deviation (SD) of three wells. Different letters (a–f) indicate the significant differences between groups (*p* < 0.05).

In addition, we found that the administration of MAPK and AKT-specific inhibitors (10 µM) (SB203580: JNK inhibitor, SP600125: p38 inhibitor, PD98059: MEK inhibitor, and LY294002: PI3K inhibitor) significantly (*p* < 0.05) restored the UVB-mediated abnormal expression of photoaging-related genes (*MMP-1a, MMP-3, COL1A1, MCP-3, IL-8* and *IL-6*) in NIH-3T3 cells (Figure 8). Interestingly, 5 uM of KFD showed an equal extent of efficacy as a 10 uM of MAPK or AKT inhibitor in restoring the UVB-mediated photoaging genes in NIH-3T3 cells (Figure 8). These results suggest that MAPKs and AKT play critical roles in KFD-mediated anti-photoaging activity by restoring the UVB-induced abnormal expression of photoaging-related genes.

### 3.7. ROS Play a Key Regulatory Role in KFD-Mediated Anti-Photoaging Events

**Figure 9:**
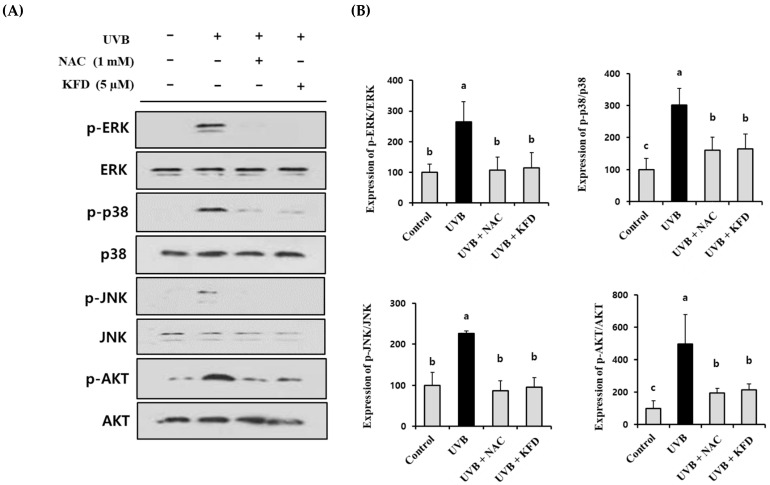
UVB-induced activation of MAPKs and AKT was restored by administration of ROS inhibitor (NAC) as well as kaempferide (KFD). NIH-3T3 cells were irradiated with UVB in the presence or absence of either ROS inhibitor: NAC (1mM) or KFD (5 µM). The expression of (**A**) each protein was estimated by Western blotting. (**B**) The density of each band was estimated by the ImageJ program and tabulated. Representative results of at least three independent experiments are shown. All data are expressed as the mean ± standard deviation (SD) of three wells. Different letters (a–c) indicate the significant differences between groups (*p* < 0.05).

Since we found that KFD is able to attenuate the UVB-induced ROS generation (Figure 5) as well as the activation of MAPKs and AKT (Figure 7), we investigated the direct connection between ROS generation and photoaging processes in UVB-irradiated NIH-3T3 cells. As shown in Figure 9, the UVB-induced phosphorylation of MAPKs and AKT was significantly (*p* < 0.05) ameliorated by an ROS inhibitor (NAC), and KFD treatment suggests that ROS has a critical regulatory role on MAPKs and AKT over phosphorylation in UVB-mediated photoaging processes. In addition, we also found that the abnormal expression of photoaging-related genes such as *COL1A1*, *MMP-1a*, *MCP-3*, *IL-6*, and *IL-8* was significantly (*p* < 0.05) restored by NAC (1 mM) treatments (Figure 8). Furthermore, 5 µM of KFD showed an equal extent of efficacy as 1mM of ROS inhibitor (NAC) in restoring the UVB-mediated expression of photoaging genes in NIH-3T3 cells (Figure 8). Collectively, these results suggest that KFD could effectively prevent the UVB-mediated photoaging processes through regulating the ROS/MAPKs and ROS/AKT signaling pathways.

## 4. Discussion

In recent years, as the elderly population has increased in many countries, skin aging induced by exposure to UV rays has led to a gradual increase in social interest and has been recognized as a major cause of geriatric diseases [31]. Therefore, there is a growing demand for more effective agents with fewer side effects than synthetic anti-photoaging compounds.

Natural agents have received much attention in the development of anti-photoaging agents because of their low cost, safety and oral bioavailability [32]. In addition, further studies have identified their individual active constituents that possess anti-photoaging activity. Since most plants biosynthesize various kinds of polyphenols as a metabolic intermediate, the major polyphenols of each medicinal plant have been isolated and targeted to investigate their medicinal activities. Previous studies have suggested that several flavonoids, a kind of polyphenol, have exhibited strong anti-photoaging activity, such as delphinidin, genistein, phloretin and salidroside [33,34,35,36]. Interestingly, in our previous study, water extracts of *Alpinia officinarum* rhizome (WEAOR) were first revealed to have strong anti-photoaging activity, and kaempferide, a flavonoid, was determined to be a major constituent of WEAOR [30]. Thus, as follow-up research, the anti-photoaging activity of kaempferide and its underlying molecular mechanism was investigated in this study. Kaempferide is an *O*-methylated flavonoid that has shown beneficial effects against the progress of several diseases, such as cancer [26,27] and inflammation [23,25]. However, little is known about the anti-photoaging properties of kaempferide and its mechanism of action.

Many studies have identified the general photoaging progress in UVB-irradiated skin, such as the reduction of dermal collagen content and inflammatory responses. We discovered that kaempferide could attenuate UVB-mediated wrinkle formation and thickening of the epidermis. In addition, we found that kaempferide could inhibit collagen degradation by inhibiting the UVB-stimulated abnormal expression of MMPs (MMP-1a and MMP-3) and recovering the UVB-induced loss of COL1A1 in mouse skin as well as NIH-3T3 skin fibroblast cells. UVB-mediated upregulation of inflammatory cytokines, such as MCP-3, IL-8 and IL-6, was attenuated in NIH-3T3 cells. Collectively, our data provide the first evidence of the anti-photoaging activities of kaempferide.

UVB-mediated reduction of subcutaneous (SC) fat is one of the main processes behind photoaging. Since UVB cannot penetrate skin tissue, it is very hard to think that UVB directly affects the loss of SC fat. Interestingly, previous studies have suggested the role of proinflammatory cytokines, such as MCP-3, IL-8 and IL-6, in the UVB-induced loss of SC fat. The administration of blocking antibodies of proinflammatory cytokines, such as IL-6, IL-8 and MCP-3, could prevent the UV-induced reduction of lipid synthesis-related genes in skin fibroblast cells, indicating the roles of proinflammatory cytokines (MCP-3, IL-8 and IL-6) as intermediators in the UVB-stimulated reduction of SC fat [14]. In fact, similar effects were reported in the WEAOR-treated photoaging mouse model. The administration of WEAOR attenuated UVB-mediated photoaging processes, including the loss of SC fat and an increase in proinflammatory cytokines (IL-6, IL-8 and MCP-3) [27]. Park et al. also reported that *Nelumbo nucifera* leaf extracts exhibit inhibitory activity against UVB-induced reduction of SC fat and increase the expression of IL-6, IL-8 and MCP-3 [6]. Since kaempferide was detected as a major constituent of WEAOR in our previous study, we investigated the effects of kaempferide on the UVB-induced reduction of SC fat and the expression of proinflammatory cytokines. Interestingly, kaempferide was proven to inhibit the UVB-induced loss of SC fat in mouse skin, and the UVB-mediated overproduction of proinflammatory cytokines (IL-6, IL-8 and MCP-3) was attenuated by kaempferide treatment. Taken together, our results suggest that kaempferide could effectively attenuate the UVB-induced reduction of SC fat by regulating the UVB-stimulated expression of SC fat intermediators, such as MCP-3, IL-8 and IL-6.

Many studies have reported that the generation of reactive oxygen species (ROS) by UVB exposure promotes the phosphorylation of mitogen-activated protein kinases (MAPKs) and AKT, which induces photoaging by regulating the expression of genes related to collagen degradation such as COL1A1 and MMPs. Therefore, the development of natural agents that are able to attenuate the activation of UVB-induced ROS-mediated signaling molecules could be used to inhibit UVB-mediated skin damage. In fact, a previous study demonstrated the anti-photoaging activity of a novel p38 MAPK inhibitor: the oral administration of SB242235, a p38 MAPK inhibitor, effectively attenuated UVB-induced skin inflammation in SKH-1 mice, which is a major step forward in photoaging [13]. In our study, we discovered that kaempferide (KFD) could block UVB-mediated ROS production as well as over-phosphorylation in NIH-3T3 skin fibroblast cells. The administration of KFD to UVB-irradiated NIH-3T3 skin fibroblast cells restore the UVB-mediated abnormal expression of photoaging-related genes. In addition, by comparing the anti-photoaging efficacy of kaempferide with that of MAPK and AKT inhibitors, we found that 5 µM of kaempferide has equivalent anti-photoaging activity to 10 μM of MAPK and AKT inhibitors. Furthermore, since we found that UVB-induced MAPKs and AKT phosphorylation was attenuated by ROS inhibitor (NAC), ROS could be a direct target in the anti-photoaging activity of KFD. Taken together, our data indicate that the anti-photoaging activities of kaempferide could be related to the attenuation of UVB-induced ROS-mediated signaling pathways.

## 5. Conclusions

In this study, the anti-photoaging properties of KFD, which is a flavonoid, were first evaluated. We found that KFD administration effectively attenuated wrinkle formation, epidermal thickening and collagen reduction in mouse skin. In addition, the UVB induced abnormal expression of collagen synthesis-related genes (COL1A1 and MMPs), and inflammation-related genes (MCP-3, IL-8 and IL-6) were attenuated by KFD treatments in mice and NIH-3T3 skin fibroblast cells. These anti-photoaging effects of KFD could be the result of attenuating intracellular ROS-mediated signaling pathways, which are activated by UVB irradiation. Collectively, our data strongly suggest that KFD could be developed as a potential anti-photoaging agent.

## Figures and Tables

**Table 1:** List of primers.

Gene	Forward	Reverse	Strain/Accession Number
IL-8	5′-TCCAATTCGGGAGACCTCTA-3′	5′-TAGGCATCACTGCCTGTCAA-3′	Mouse/NM_011339.2
IL-6	5′-ACAACCACGGCCTTCCCT-3′	5′-AGCCTCCGACTTGTGAA-3′	Mouse/M20572.1
MCP-3	5′-ATAGCCGCTGCTTTCAGCAT-3′	5′-CTTCCCAGGGACACCGACTA-3′	Mouse/BC061126.1
MMP-1a	5′-ACTTTCCAGCCAGGCCCA-3′	5′-CACTGCTGTTGGTCCACGT-3′	Mouse/NM_032006.3
MMP-3	5′-TTCTGGGCTATACGAGGGCA-3′	5′-CTTCTTCACGGTTGCAGGGA-3′	Mouse/NM_010809.2
COL1A1	5′-CCACAATGGCACGGCTGT -3′	5′-AAAGCACAGCACTCGCCC-3′	Mouse/NM_007742.4
GAPDH	5′-AAGCTGTGGCGTGATGGC-3′	5′-TGACCTTGCCCACAGCCT-3′	Mouse/GU214026.1

## Data Availability

The data that support the results in this study are available from the corresponding author on reasonable request.

## References

[1] Decean H., Fischer-Fodor E., Tatomir C., Perde-Schrepler M., Somfelean L., Burz C., Hodor T., Orasan R., Virag P. (2016). Vitis vinifera seeds extract for the modulation of cytosolic factors BAX-alpha and NF-kB involved in UVB-induced oxidative stress and apoptosis of human skin cells. Clujul Med..

[2] Gilaberte Y., Gonzalez S. (2010). Update on photoprotection. Actas Dermosifiliogr..

[3] Fisher G.J., Kang S., Varani J., Bata-Csorgo Z., Wan Y., Datta S., Voorhees J.J. (2002). Mechanisms of photoaging and chronological skin aging. Arch. Dermatol..

[4] Gilchrest B.A. (1989). Skin aging and photoaging: An overview. J. Am. Acad. Dermatol..

[5] Kim E.J., Jin X.J., Kim Y.K., Oh I.K., Kim J.E., Park C.H., Chung J.H. (2010). UV decreases the synthesis of free fatty acids and triglycerides in the epidermis of human skin in vivo, contributing to development of skin photoaging. J. Dermatol. Sci..

[6] Park K.M., Yoo Y.J., Ryu S., Lee S.H. (2016). Nelumbo Nucifera leaf protects against UVB-induced wrinkle formation and loss of subcutaneous fat through suppression of MCP3, IL-6 and IL-8 expression. J. Photochem. Photobiol. B.

[7] Wolf P., Yarosh D.B., Kripke M.L. (1993). Effects of sunscreens and a DNA excision repair enzyme on ultraviolet radiation-induced inflammation, immune suppression, and cyclobutane pyrimidine dimer formation in mice. J. Investig. Dermatol..

[8] Zaidi M.R., Day C.P., Merlino G. (2008). From UVs to metastases: Modeling melanoma initiation and progression in the mouse. J. Investig. Dermatol..

[9] Kang W., Choi D., Park T. (2019). Dietary Suberic Acid Protects Against UVB-Induced Skin Photoaging in Hairless Mice. Nutrients.

[10] Myung D.B., Han H.S., Shin J.S., Park J.Y., Hwang H.J., Kim H.J., Ahn H.S., Lee S.H., Lee K.T. (2019). Hydrangenol Isolated from the Leaves of Hydrangea serrata Attenuates Wrinkle Formation and Repairs Skin Moisture in UVB-Irradiated Hairless Mice. Nutrients.

[11] Pittayapruek P., Meephansan J., Prapapan O., Komine M., Ohtsuki M. (2016). Role of Matrix Metalloproteinases in Photoaging and Photocarcinogenesis. Int. J. Mol. Sci..

[12] Amaro-Ortiz A., Yan B., D’Orazio J.A. (2014). Ultraviolet radiation, aging and the skin: Prevention of damage by topical cAMP manipulation. Molecules.

[13] Kim A.L., Labasi J.M., Zhu Y., Tang X., McClure K., Gabel C.A., Athar M., Bickers D.R. (2005). Role of p38 MAPK in UVB-induced inflammatory responses in the skin of SKH-1 hairless mice. J. Investig. Dermatol..

[14] Kim E.J., Kim Y.K., Kim J.E., Kim S., Kim M.K., Park C.H., Chung J.H. (2011). UV modulation of subcutaneous fat metabolism. J. Investig. Dermatol..

[15] Lawrence K.P., Douki T., Sarkany R.P.E., Acker S., Herzog B., Young A.R. (2018). The UV/Visible Radiation Boundary Region (385-405 nm) Damages Skin Cells and Induces “dark” Cyclobutane Pyrimidine Dimers in Human Skin in vivo. Sci. Rep..

[16] Cao W.Q., Zhai X.Q., Ma J.W., Fu X.Q., Zhao B.S., Zhang P., Fu X.Y. (2020). Natural borneol sensitizes human glioma cells to cisplatin-induced apoptosis by triggering ROS-mediated oxidative damage and regulation of MAPKs and PI3K/AKT pathway. Pharm. Biol..

[17] Ding Y., Jiratchayamaethasakul C., Lee S.H. (2020). Protocatechuic Aldehyde Attenuates UVA-Induced Photoaging in Human Dermal Fibroblast Cells by Suppressing MAPKs/AP-1 and NF-kappaB Signaling Pathways. Int. J. Mol. Sci..

[18] Jung J.M., Choi J.K., Kwon O.Y., Lee S.H. (2022). Anti-Photoaging Activity of Scutellaria barbata D. Don (Family Lamiaceae) on Ultraviolet B-Irradiated NIH-3T3 Skin Fibroblast and SKH-1 Hairless Mouse. Molecules.

[19] Ren J., Su D., Li L., Cai H., Zhang M., Zhai J., Li M., Wu X., Hu K. (2020). Anti-inflammatory effects of Aureusidin in LPS-stimulated RAW264.7 macrophages via suppressing NF-kappaB and activating ROS- and MAPKs-dependent Nrf2/HO-1 signaling pathways. Toxicol. Appl. Pharm..

[20] Hwang Y.P., Kim H.G., Han E.H., Choi J.H., Park B.H., Jung K.H., Shin Y.C., Jeong H.G. (2011). N-Acetylglucosamine suppress collagenases activation in ultraviolet B-irradiated human dermal fibroblasts: Involvement of calcium ions and mitogen-activated protein kinases. J. Dermatol. Sci..

[21] Kwon K.R., Alam M.B., Park J.H., Kim T.H., Lee S.H. (2019). Attenuation of UVB-Induced Photo-Aging by Polyphenolic-Rich Spatholobus Suberectus Stem Extract Via Modulation of MAPK/AP-1/MMPs Signaling in Human Keratinocytes. Nutrients.

[22] Lee J.E., Oh J., Song D., Lee M., Hahn D., Boo Y.C., Kang N.J. (2021). Acetylated Resveratrol and Oxyresveratrol Suppress UVB-Induced MMP-1 Expression in Human Dermal Fibroblasts. Antioxidants.

[23] Honmore V.S., Kandhare A.D., Kadam P.P., Khedkar V.M., Sarkar D., Bodhankar S.L., Zanwar A.A., Rojatkar S.R., Natu A.D. (2016). Isolates of Alpinia officinarum Hance as COX-2 inhibitors: Evidence from anti-inflammatory, antioxidant and molecular docking studies. Int. Immunopharmacol.

[24] Bian Q.Y., Wang S.Y., Xu L.J., Chan C.O., Mok D.K., Chen S.B. (2013). Two new antioxidant diarylheptanoids from the fruits of Alpinia oxyphylla. J. Asian Nat. Prod. Res..

[25] Tang H., Zeng Q., Ren N., Wei Y., He Q., Chen M., Pu P. (2021). Kaempferide improves oxidative stress and inflammation by inhibiting the TLR4/IkappaBalpha/NF-kappaB pathway in obese mice. Iran J. Basic. Med. Sci..

[26] Matsuda H., Nakashima S., Oda Y., Nakamura S., Yoshikawa M. (2009). Melanogenesis inhibitors from the rhizomes of Alpinia officinarum in B16 melanoma cells. Bioorg. Med. Chem..

[27] Tasdemir D., Kaiser M., Brun R., Yardley V., Schmidt T.J., Tosun F., Ruedi P. (2006). Antitrypanosomal and antileishmanial activities of flavonoids and their analogues: In vitro, in vivo, structure-activity relationship, and quantitative structure-activity relationship studies. Antimicrob. Agents Chemother..

[28] Kumkarnjana S., Suttisri R., Nimmannit U., Sucontphunt A., Khongkow M., Koobkokkruad T., Vardhanabhuti N. (2019). Flavonoids kaempferide and 4,2′-dihydroxy-4′,5′,6′-trimethoxychalcone inhibit mitotic clonal expansion and induce apoptosis during the early phase of adipogenesis in 3T3-L1 cells. J. Integr. Med..

[29] Tang H., Zeng Q., Tang T., Wei Y., Pu P. (2021). Kaempferide improves glycolipid metabolism disorder by activating PPARgamma in high-fat-diet-fed mice. Life Sci..

[30] Jung J.M., Kwon O.Y., Choi J.K., Lee S.H. (2022). Alpinia officinarum Rhizome ameliorates the UVB induced photoaging through attenuating the phosphorylation of AKT and ERK. BMC Complement. Med. Ther..

[31] Travers J.B., Spandau D.F., Lewis D.A., Machado C., Kingsley M., Mousdicas N., Somani A.K. (2013). Fibroblast senescence and squamous cell carcinoma: How wounding therapies could be protective. Dermatol. Surg..

[32] Afaq F. (2011). Natural agents: Cellular and molecular mechanisms of photoprotection. Arch. Biochem. Biophys..

[33] Mao G.X., Xing W.M., Wen X.L., Jia B.B., Yang Z.X., Wang Y.Z., Jin X.Q., Wang G.F., Yan J. (2015). Salidroside protects against premature senescence induced by ultraviolet B irradiation in human dermal fibroblasts. Int. J. Cosmet. Sci..

[34] Shin S., Kum H., Ryu D., Kim M., Jung E., Park D. (2014). Protective effects of a new phloretin derivative against UVB-induced damage in skin cell model and human volunteers. Int. J. Mol. Sci..

[35] Wang Y.N., Wu W., Chen H.C., Fang H. (2010). Genistein protects against UVB-induced senescence-like characteristics in human dermal fibroblast by p66Shc down-regulation. J. Dermatol. Sci..

[36] Allemann I.B., Baumann L. (2009). Botanicals in skin care products. Int. J. Dermatol..

